# What is in a Meter? A Qualitative Exploration into the Implementation of Electricity Metering Across Mumbai Communities Using Normalisation Process Theory

**DOI:** 10.1007/s43477-022-00059-y

**Published:** 2022-10-11

**Authors:** Gillian Waller, Tracey Crosbie, Dorothy Newbury-Birch, Santanu Bandyopadhyay, Dana Abi Ghanem, Arnab Jana, Gobind G. Pillai, G. S. Krishna Priya, Ahana Sarkar, Neenu Thomas, Parisa Diba, Andy Divers

**Affiliations:** 1 School of Social Sciences, Humanities and Law, Teesside University, Middlesbrough, TS1 3BA UK; 2 Department of Energy Science and Engineering, Indian Institute of Technology Bombay, Mumbai, India; 3 Centre for Urban Science and Engineering, Indian Institute of Technology Bombay, Mumbai, India; 4 Centre for Sustainable Engineering, Teesside University, Middlesbrough, TS1 3BA UK; 5 KRVIA, University of Mumbai, Mumbai, India

**Keywords:** Metering, Meters, Electricity, Implementation, Normalisation Process Theory, Qualitative

## Abstract

Metering is fundamental in the efficient operation of electricity networks, as meters facilitate controlled usage and improve health and well-being. However, across the Global South, meters have often been found to be lacking or not fit for purpose. Therefore, this study sought to determine residents’ perceptions and access to electricity metering across a community in Mumbai, with the goal of developing recommendations to support the implementation of meters in the future. Fifty semi-structured interviews were conducted by phone, with participants from different areas and socioeconomic classes, within Greater Mumbai. The sample consisted of 20 low-income, 20 middle-income, and 10 high-income participants. The Normalisation Process Theory (NPT) was used to inform the interview schedule and to organise the thematic analysis. Meter accessibility and location was variable across the participant groups, as was the education and awareness of metering technology. Socio-political factors were found to directly affect the use of meters, specifically in the low-income group. The high cost associated with metering was a prominent finding; with a preconception that introducing meters would only increase utility expenditure. Future work should focus around ensuring meters are easy to use, practical and accessible to all residents and supporting education programmes around how to use a meter and how they can reduce utility expenditure. The cost of meters should also be investigated, to establish that the costs, associated with introducing new meters, are not passed disproportionately to consumers.

**Supplementary Information:**

The online version contains supplementary material available at 10.1007/s43477-022-00059-y.

## Background

Meters can have a plethora of different functions, but are often described as gateway technologies as they are commonly used to control access to utilities, such as clean drinking water and electricity, across different economic classes (Jana et al., 2021). In recent times, electricity metering has been identified as being fundamental in ensuring the efficient operation and planning of electricity networks, as they facilitate controlled usage and promote better health and well-being, by delivering safe and regulated products (Boyle et al., 2013; Jana et al., 2021). Meters have successfully been incorporated into the regulation of utilities in many countries across the world, with significant benefits experienced (Jana et al., 2021; Boyle et al., 2013; Vesal et al., 2018; Dresner & Eskins, 2006; Harutyunyan, 2015). The recognition of the advantages of metering has led both national and international agencies and governments encouraging the introduction of ‘smarter’, more comprehensive metering in the respective sectors.

However, in India, meters are often ineffective or not fit for purpose (Kumar, 2010). Over several decades, legislative and policy measures have been introduced to enhance the performance of the power sector in India. This has been spear-headed by the introduction of a series of five-year plans, which have chronologically been released with the key intentions to improve the supply and distribution (Priya et al., 2022). The most recent is the 12th and final five-year plan, which was first introduced in 2012 (Ministry of Power, 2020). The introduction of metering was proposed in these plans, with the 12th plan being amended in 2019 to include an increased focus on smart metering, following the successes in other countries. Nevertheless, the current smart metering legislation remains in its infancy in India and with the transition to smart meters still a work in progress, more evidence is needed to inform and develop the sector.

Generally, in Mumbai, the focus of this study, the utility distribution companies are responsible for implementing metering. There are three companies supplying electricity: two private companies and one public sector enterprise (Priya et al., 2022). Practically, 100% of the connections should be metered and when there are discrepancies, it tends to be a result of two or more consumers using the same meter (Jana et al., 2021; Priya et al., 2022). There are often cost implications associated with ineffective metering. For example improper billing and meter tampering, and inequities are frequently observed across different socioeconomic groups, with lower income groups being the most affected (Priya et al., 2022).

The context of metering in India follows international development discourse on the need for new and improved metering to ensure better management of electricity provision in the country, by instituting new methods of consumer accountability and cost recovery (International Energy Agency, 2021). There are technical improvements to be made on the provision infrastructure, as well as societal tools for responsibility and development (Kumar, 2019). As Coleman (2014) argues, such technologies are imbued with notions of progress and civility that their meanings and their implications should be a matter of concern (Kumar, 2021), which ultimately lays the foundations for this current study.

The ‘What is in a Meter?’ study was proposed to explore the current metering climate in Mumbai, India, and how the access to essential services can be re-configured within metering technologies and implemented in the rapidly growing cities.^1^ The study is split into three distinct components, called work packages, each exploring different facets of metering technology, but with the overarching aim to inform the implementation and use of metering in ways that engender social inclusion, environmentally sensitive consumption patterns and reduce health inequalities.

This paper focuses on the findings from work package 3, which was a qualitative investigation focused on exploring residents’ perceptions, access, and knowledge of electricity meters across different socioeconomic groups, within a community. It also sought to determine what facilitators and barriers exist in relation to developing and implementing improved metering technology in the future, from the perspective of the service users. There has been a comprehensive body of quantitative work looking at the prevalence and the introduction of different types of electricity meters across the world. However, this study set out to build upon the growing body of work exploring the perceptions and access to metering in the developing world from a qualitative perspective. The currently available qualitative evidence in this area is not as extensive and there have been fewer examples of work exploring service users’ thoughts around the use of meters, which this study sought to address. By incorporating a qualitative investigation into the project, it will shape future practice by recognising the concerns of service users and the facilitators and barriers associated with residents engaging with metering.

One of anticipated outcomes of this investigation was to determine how new metering technologies could be implemented more widely in the future. There has been a growing call for global health to focus on the implementation of new interventions, due to the recognition that effective implementation of a new technology, can have the potential to increase its success and significantly improve resource-poor settings (Yapa et al., 2018; Ridde et al., 2020). This study aims to facilitate meter implementation by providing a novel perspective to the metering technology setting, in which the currently available, implementation evidence base is limited. In addition, studies exploring the implementation of global health interventions have not been widely informed by implementation theories and analytic frameworks (Ridde et al., 2020). Employing the use of an implementation theory can assist in establishing the determinants of implementation, whilst considering the specific factors that have the potential to affect an implementation process (Nilsen, 2015; Sarkies et al., 2021). Therefore, to add to the existing evidence base, the study’s data collection and analysis were informed by an implementation theory, the Normalisation Process Theory.

## Aim

The aim of this study was to explore the access to electricity metering, across differing socioeconomic groups, within a community and to determine how the technology can be introduced more widely in the future by determining the barriers and facilitators to implementation.

## Methods

### Study Design

The study design was a descriptive qualitative design, with semi-structured interviews chosen as the primary data collection method. We selected semi-structured interviews, as although it was imperative to ask participants specific questions around their current living situations and environment, it was also important to ensure participants could talk freely and informally about their own insights and experiences around metering (Longhurst, 2003). Therefore, by developing a semi-structured interview schedule, it allowed a pathway of questioning to be formulated, which also had an element of flexibility depending on the responses provided by a participant.

### Setting

The primary research setting was different communities within Greater Mumbai. Mumbai has a very diverse population, consisting of multiple religions, castes, and economic classes. As we anticipated heterogeneity across participants from different socioeconomic groups; the sample was selected by differences in income level. We identified income level as the main sampling criteria as the income level of a participant largely determines the type of accommodation available to them.

Individuals within lower income groups often inhabit slum houses or slum rehabilitation projects (vertical slums). Given that more than 50% of Mumbai's population lives in slums, the state government in 1975 set up the Slum Rehabilitation Authority (SRA) (Nallathiga et al., 2019). The objective was to improve the living conditions of the low income group by providing alternative housing, either on site, or at a different location. Studies however, show that while there is some improvement in physical infrastructure (such as sanitation facilities and solid waste management options), these projects have often failed to provide social infrastructure such as access to affordable education, health care, and recreation (Nallathiga et al., 2019). In addition, the displacement associated with rehousing often results in livelihood loss as well. Individuals within medium income groups typically rent or own houses in apartment complexes, usually in the suburbs, while those with higher income inhabit larger, more luxurious houses in desirable areas. The researcher conducting the interviews was located in Kerala and all interviews were conducted via phone. Kerala is a southwestern coastal state of India (Noble, 2022). It is a relatively small state, as it constitutes only 1% of the total area of India, however it is one of the most densely populated states (Noble, 2022).

### Participants

The proposed sample was to include between 30 and 60 residents across different communities. We proposed this large range of participants as the sample obtained was dependent on the success of the recruitment processes within the COVID-19 climate, as we anticipated this would make recruitment more difficult. We also wanted to ensure that the later data collection was able to generate new findings before reaching a state of data saturation (Baker & Edwards, 2012; Fusch & Ness, 2015). The sample was categorised by socioeconomic status. We sought approximately 10 to 20 interviewees from a low-income area or group (LIG), 10 to 20 interviewees from a middle-income area (MIG) and 10 to 20 interviewees from a high-income area (HIG), as we believed this would be an appropriate starting number of participants to explore the aims of the study. Income categories often vary by state, but typically those with income under 3lakh Rs per annum are considered lower income. However, in a metro city like Mumbai, the levels for survival are higher. Therefore, for the purpose of this study, those with under 5L Rs annual income per household were considered to be in the LIG category, and anything above 30 L Rs per annum was considered HIG. Anything in between was considered MIG. Eligible participants were adults aged 18 years or over, who were able to provide their informed consent to take part in an interview about how they accessed electricity. We proposed that if this sample was either identified as being too large, with no new information being provided in later interviews, or if it was not of sufficient size to reach a state of data saturation, then it would be reduced or expanded as necessary.

### Sampling and Recruitment

The study required recruitment of participants of various socioeconomic strata (defined by income levels) and thus a snowball sampling technique was adopted (Biernacki & Waldorf, 1981; Goodman, 1961). Snowball sampling was used to recruit LIG and MIG participants by identifying a familiar service provider or individual, who was known to the researcher, (examples included shopkeepers, maids, and taxi drivers) and they were invited to take part in the study or recommend others within their peer group, such as friends, colleagues, neighbours. Similarly, university faculty members introduced contacts, expanding into wider peer groups which formed the HIG sample. Given the COVID-19 pandemic and the intensity with which Mumbai was affected, it was not possible to conduct the interviews in person. Establishing trust was difficult, especially when sensitive topics such as electricity billing and metering were involved, particularly in the lower income group. Snowball sampling was used to help minimise this lack of trust, as researchers were introduced to the participant by someone known within the participant’s peer group.

The sample contained both male and female participants and the interviews were conducted within mixed gender households. The research team did not impose any constraints on gender and spoke to whichever member of the family was available for the call. Participants were initially called by the researcher to introduce the study, obtain consent and to fix an appropriate time for an interview to take place.

### Data Collection

We used the Normalisation Process Theory (NPT) to inform and structure the interview questions, to ensure a comprehensive overview of the factors affecting the future implementation was obtained (Waller et al., 2017). The Normalisation Process Theory (NPT) is an implementation theory developed in the context of healthcare innovations and largely focuses on understanding the implementation, embedding and integration of new technologies and organisational innovations (May et al., 2009; May & Finch, 2009; May et al., 2011). It consists of four theoretical constructs that can be considered when assessing a practice: Coherence which refers to individuals making sense of a new technology, Cognitive Participation which considers the relational work that individuals do to build and sustain a new technology, Collective Action which assesses the operational work needed to implement a new technology and Reflexive Monitoring which is the appraisal individuals can do to assess and understand how a new technology affects them and others around them (May et al., 2009; May & Finch, 2009; May et al., 2011).

We developed the interview schedule using the four main NPT constructs, to propose questions that would effectively explore the implementation of metering technology, whilst addressing the outcomes of the study. We selected the NPT because existing literature has shown it to be dynamic and that it can be used fluidly to synthesise research findings and identify knowledge consistencies and gaps regarding implementation determinants (Mair et al., 2012; O'Reilly et al., 2017). It has not previously been used within the metering technology field, prior to the completion of this project. However, as it has proved its value across different settings outside of healthcare (Waller et al., 2017), it was adopted for use within this study and hence offers another novel topic area for the application of NPT.

The interview schedule has been included as a supplementary file and includes the NPT annotations to document how the interview questions relate to the different NPT constructs. Researchers used the same interview schedule for all interviews to maintain rigour and included the same relevant prompts to support further enquiry with participants. We ensured that there were no leading questions in the interview schedule, and participants were encouraged to talk freely about the topic to express their thoughts. We also conducted pilot interviews, with members of the different communities, to test the early versions of the questions, to ensure that the schedule was fit for purpose, that all relevant areas were covered and that the questions were easily understandable.

Given the COVID-19 pandemic, obtaining consent sheets which were physically signed was not possible. Therefore, we provided participants, who expressed an interest in being interviewed, with a verbal explanation of the project over the phone and it was relayed that any data related to personal identity (name, address, etc.) would be removed from the transcript. We obtained verbal consent to take part by reading out the consent form over the phone, prior to starting the interview. All prospective participants were fully informed about how their data would be used and the proposed dissemination routes. It was found that participants generally preferred to take part in an interview at home, outside of work hours and all interviews were conducted remotely by phone.

We audio recorded all interviews for analysis purposes and these were transcribed verbatim, with all personal or identifiable information removed. Most interviews were carried out over several months by one researcher (Female, Middle Income Group). The interviews were primarily done in English, Hindi, and Malayalam, all of which the researcher is fluent in. In a few cases (less than 5 in total), other regional languages were involved (Tamil and Marathi), two other researchers (Female, Middle Income group) conducted these interviews to ensure a greater range of participants could be included in the sample. The two additional interviewers were briefed on the study and were well versed in conducting interviews. Any interviews that were not undertaken in English, were translated into English to ensure that the researchers in the UK, conducting the data analysis, could consider all of the data collected together. All data, including audio recordings and interview transcripts, were stored securely on password protected devices and were only shared within the research team via secure file sharing services.

### Data Analysis

We analysed the interview data using a thematic analysis approach, as detailed by Clarke and Braun (Clarke & Braun, 2017) to ensure consistency. Initially we assessed all of the interview data to obtain any general comments or similarities appearing in the data (Clarke & Braun, 2017; Elliott, 2018). As NPT was used in the construction of the interview questions, we used NPT again during this data familiarisation stage to identify areas of the implementation process that were widely discussed by participants and identify areas which received less attention (May et al., 2015). The validated NPT toolkit was used for this stage as it unpacks each construct into four statements creating 16 sub-constructs and hence is the most detailed level of the NPT constructs (May et al., 2015). Using the NPT toolkit allowed the analysis to be informed and structured by NPT and enabled a more in-depth analysis to be undertaken.

We then coded the interview data by assigning short words or phrases which represented the meaningful responses provided by participants. Two researchers coded all of the data independently to ensure reliability and that no relevant responses had been overlooked or misconstrued. We then assimilated the codes generated by both researchers and these were organised into overarching themes (Clarke & Braun, 2017). By grouping together analogous codes, specific areas, or themes emerging from the data were able to be identified (Clarke & Braun, 2017). This facilitated the development of coding grids, which organised the codes by theme and NPT sub-construct. We conducted a comparative style analysis, as any emerging themes from the early data were utilised when coding later interview transcripts, until we recognised that data saturation had been reached (Fusch & Ness, 2015).

### Ethics

An application for ethical approval for this study was submitted to the Teesside University School of Computing, Engineering and Digital Technologies Ethics and Research Governance Committee and it was approved on the 23/06/2020.

## Results

Fifty interviews were conducted; 20 interviews conducted with low-income participant groups (LIG), 20 interviews were conducted with middle- income participants (MIG), and in the case of high-income participants (HIG), data saturation was reached after 10 participants. Table 1 displays the numbers of each participant by gender, language, and location.

**Table 1:** Table of participant demographic information

	LIG	MIG	HIG
Gender			
Male	11	10	5
Female	9	10	5
Language			
Marathi MT	1		
Hindi HI	7	1	
Malayalam ML	9		
Tamil TM			
English	3	19	10
Location			
Powai PWI	18	12	
Borivalli (BLI)		2	
Mahim (MHM)		2	
Grant Road (GTR)		1	
Dahisar (dhr)		1	
Mulund (MUL)		2	
Andheri (AD)	1		
Chandivali (CH)	1		

Table 2, included as a supplementary document, presents all of the codes that were generated, arranged by the four NPT constructs. Following the completion of the thematic analysis, all of the codes were assimilated to create four main themes. The four themes were: Meter’s Location and Area Demographics, Access to Meters, Cost and Awareness and Education. The following sections will go on to present these in more detail with illustrative quotes from the participants.

## Area Demographics

The participants across the three socioeconomic groups talked about their area and the respective positives and negatives as part of the background questions. The HIG participants generally lived closer to high quality amenities and services and were more likely to already have meters in place in their accommodation. The LIG participants, and specifically those residents living in slums, were found to have a more limited access to local services and were much less likely to have meters installed or were relying on outdated metering systems which were not properly regulated or controlled. Chawls are extremely prevalent in Mumbai and refer to low-cost row houses, that accommodate families in small individual apartments, which commonly share facilities, such as bathrooms.

As one HIG participant stated:

it all depends on the area where unmetered stuff and also for example, if you are, if you go to some of these areas, which is that which are I wouldn't say exactly slum you know, but, but there are chawl systems in Mumbai a lot of areas where the middle class or lower middle class pays right, which is which is a chawl system or very old buildings, you know that there is no proper society. There is no proper, there is no kind of meter supervision which needs to be done by the society. (HIG-001).

The presence of utility meters was often a selling point when participants were searching for a new property. This relates to NPT’s Coherence construct, with a participant being able to see the value in having a meter in their living arrangements. An example of this can be seen by participant HIG-003, who reflected upon the fact that meters were often considered as part of an individual’s decision to move into a new area:

It's actually demand for housing, the people who live here are all quite well off. So for somebody who wants to pick up a house here, if a builder does not have this facility. They're not picking up the house here. So it is a demand and supply situation where the builder is forced to view all these amenities properly. Otherwise, people won't move into this this thing. (HIG-003)

Although it was deemed as important to have meters in place within housing, often the residents’ access to meters was variable across the three participant groups, which will be explored in the next section.

## Access to Meters

The findings around the variable availability and access to meters fits within NPT’s Cognitive participation construct, as it allows the consideration of whether a participant is supporting or ‘buying into’ a practice. Meter accessibility was variable across the three groups. Those living in rented accommodation were at the jurisdiction of their landlord. Although some landlords appeared to be diligent at ensuring tenants had access to the meters, others were not as forthcoming. This led to issues for those residents who wanted to keep a track of their consumption and hence reduce their support and ‘buy-in’ for meters as they were not able to access them. The majority of LIG participants interviewed lived in rented housing and many were unable to see their meters and hence had to simply pay the bill they were presented with. According to one participant, “We don’t see the meter. Instead, we pay 200 rs every month with the bills” (LIG-013).

Not being able to access utility meters was often compounded by socio-political factors that directly affected the use of meters, specifically in the LIG. Several participants talked about their access to meters being ‘blocked’ and stated that the political and area leaders are not always upfront with housing information, leading to limited access to their meters or areas with no meters being implemented at all. As outlined by a MIG participant:

It seems slum people are under the impression that if they have a chawl meter, they’re going to have to pay more(…) I think they are mis-informed by political leader, there is information also, people are afraid of their leader because they might be under a lot of ah, they have to survive. (MIG-002)

Further social challenges affecting implementation included a LIG participant reporting that their access to their meter, within their accommodation, was limited due to the need for increased security. This was enforced to avoid individuals tampering with the meters, which had previously led to individuals being able to foster illegal connections. As a LIG participant highlighted, “But some things do happen. People may pull an illegal connection. We now keep the meter locked. There are always a few people who do a bit of mischief. So if the security can be improved” (LIG-020).

The access to meters was also affected by the COVID-19 pandemic. Participants across all income groups were not able to access their meters during the pandemic, resulting in bills being sent without meter readings. In some cases planned meter improvements did not occur due to the pandemic. One participant shared the following: “it’s a really old structure and needs to get into redevelopment. So that's not happened. And we're all waiting for it to happen soon. And, I mean, because it's COVID time” (MIG-018).

## Cost

Another key theme observed within the data, and a potential barrier to implementing metering, is cost. All of the participants interviewed, across the three income groups, reflected upon the cost of energy and how metering has affected or might affect their electricity bills. There appeared to be a perception from LIG participants, that if their current meter was improved or if they had a meter installed, then the cost of their bills would significantly increase.

Cost is very high for metering. The bills are very high. That is the only problem. (…) Since [NAME REMOVED] have taken up the charge, cost has increased to 2500 INR per month. In the last 6 months during corona, my electricity bill has amounted to 11070 INR. Bills are always on higher side with metering. (LIG-001)

The notion that energy meters increased the cost of energy for householders was also found across the other participant groups. A MIG participant remarked that individuals who did not have meters in place often accessed their utilities by other illegal means, resulting in the current cost being disproportionately low:

I think the biggest concern is probably people who might not have meters is the concern that they might have to pay a lot of money for the services which they already might be getting, you know from other sources? (MIG-002)

However, there were also social cost advantages of using meters as discussed by LIG participants. One participant talked about how the meter in their shared accommodation allowed the utility bills to be shared across residents, which avoided any issues with cutting off the supply across the building, a factor that would affect all the residents: “Even if one member sometimes fails to contribute to the energy bill, the amount gets distributed among other members. So, there is no chance of disconnecting any connection” (LIG-002).

This sometimes led to feelings of resentment as some participants felt their bills were disproportionately high, but it also fostered an increased feeling of community in the LIG areas by helping individuals in times of need and struggling.

## Awareness and Education

The final theme emerging from the data relates to the participants’ level of awareness and education around meters. This included understanding around what the meters can be used for, how they can be used to control utility usage and how the bills are interpreted. These findings were indicative of NPT’s Coherence construct when understanding the importance of metering and the value it can hold in comparison to the usual practice, and the Collective Action construct around supporting the work of metering. Generally, across the participants interviewed, the lowest level of understanding of meters and understanding their bills appeared to be in the LIG participant group. Indeed, as stated by one LIG participant: “Honestly I can no longer read and understand an electricity bill. There are so many charges for various reasons and it all comes to a really huge amount” (LIG-004).

It was found that the LIG participants either did not understand what a meter was used for or found it difficult understanding their utility bills. In addition, LIG participants reported being unaware of how to use meters or whether they had one in place at all. This was exacerbated during the COVID-19 pandemic, as meters were not read at regular intervals and bills were just sent out, leading to the participants reporting they had received higher than normal bills. For one LIG participant: “During COVID time a lot of people were struggling because they did not check the meter and they just send the bill” (LIG-002).

In contrast, the MIG and HIG participants who used their meters and talked about having the most recently updated technology, reported being happy with their bills and being able to control their utility consumption. As stated by a MIG participant: “As a user it is fine like I can clearly understand what my reading is and what my usages is” (MIG-001).

Both the MIG and HIG participants however, discussed the importance of being educated around using meters and the new technology and how they worked to control consumption, as underscored in a MIG participant’s account: “People are not even though they are educated about current (…), I think people should be educated how they can save electricity” (HIG-002).

Often, participants displayed a level of disinterest in looking at their bills, or not being able to find the time to learn how their meter works, and how to interpret their bills. This was a finding observed across all three of the participant groups, with one participant in particular talking about how there was no difference between the busy lives of LIG and HIG participants and that their lack of engagement with a meter was more likely down to the differences in awareness levels.

It is the time. Because people are very busy and in business that is no difference between lower income are higher income. here all people are busy. So anyway, awareness is good only. If people have awareness they will monitor consumption there is no doubt that. (MIG-001)

This was also observed in the account of LIG-009 who reflected upon the fact that any money saved by understanding how to use a meter, would be negated by having to take time off work to increase their understanding: “Honestly, I don’t have enough time to spend on it. If I have to spend a day on it, the money that I lose when I take leave from work will be more” LIG-009.

## Discussion

The aim of this study was to explore the access to electricity metering, across differing socioeconomic groups, within a community and to determine how the technology can be introduced more widely in the future, by determining the current barriers and facilitators to implementation. The key findings from the interviews were that meter accessibility and location was variable across the participant groups, as was the education and awareness of metering technology. Socio-political factors were found to directly affect the use of meters, specifically in the low-income group. The high cost associated with metering was a prominent finding; with a preconception that implementing meters in the future would only increase utility expenditure.

It is firstly important to note that this work was conducted during the COVID-19 pandemic. The pandemic impacted on all aspects of the study and consequently, all interviews had to be conducted remotely. During the interviews, participants talked about how COVID-19 had affected meter readings and delayed improvements of their meters. This is compounded by the fact that personal consumption of electricity increased during the pandemic, as a result of the increase in remote working, as shown in recent studies (Di Mauro et al., 2021). Therefore, the importance of improving the metering infrastructure became even more salient in the changing landscape of the pandemic as this study progressed. However, with reduced resources and capacity because of COVID-19, it is likely that such improvements may become ever more challenging to implement.

From the interview findings, it is highly apparent that there needs to be an increase in the education and awareness of the benefits of metering that is comprehensive and includes individuals from all socioeconomic groups. Participants being unaware of meters and lacking an understanding of how they function, was found to decrease the perceived value of meters, leading to individuals being less likely to engage with and be interested in implementing utility metering technologies. This finding was supported in work by Jay et al., (2019), which explored metering adoption in America, as they found communicating the economic benefits to the public, would likely enhance acceptance (Jay et al., 2019). Lack of awareness and education is highly indicative of the NPT construct Coherence, as if participants lack an understanding around the practice of metering, they are unlikely to adopt or see the value in their future implementation. Therefore, a way of increasing the availability of information and meter awareness must be a target for future work.

One potential option could be understanding the plethora of advantages of adopting smart metering, discussed in-depth in the work of Van Gerwen et al. (2006), such as increased energy saving and safety of supply, as this could act as a facilitator for future implementation (Van Gerwen et al., 2006). Another relevant example, concurrent with this study’s findings, is considering the success of education around metering in South Africa. Although their implementation of metering is in similar stages to India, large-scale educational advertisement programs, around smart meters and how customers would be able to better regulate their energy use, have been advantageous in increasing awareness (Priya et al., 2022).

Another finding affecting participants’ readiness to engage with metering are deep-rooted, preconceived ideas around the cost of metering, in that high costs would be passed on to the consumer, and thus exacerbating the fear that introducing meters would increase an individual’s utility expenditure. Rausser et al. supported this finding around cost as they reported that the implementation of smart meters, in the UK, was facilitated by conveying a reduction in the cost of meters and the associated costs (Rausser et al., 2018). Again, considering metering in South Africa; the installation of four million prepaid meters reduced the billing infrastructure and manpower, therefore reducing the meter reading cost and achieving better revenue recovery (Priya et al., 2022). In addition, Jack and Smith reported that this reduction in cost in South Africa, would likely result in a reduction in the profitability gap between the richest and poorest customers (Jack & Smith, 2016). Reducing the profitability gap would be of significant benefit when considering the future implementation of metering in Mumbai, due to the differences observed across the different socioeconomic groups.

The cost of metering, combined with the lack of awareness and education, were also influenced by socio-political factors. Many interviewees reported that community leaders and local government officials were embroiled in corruption contributing to a lack of transparency in relation to costs of utilities. Future work should unpack and explore the social factors around the cost implications and how to ensure that implementing new meters does not have a significant cost implication to consumers. The implementation of smart metering can be associated with higher investment costs and policy measures are needed to ensure that these do not affect low-income groups negatively. Alternatively, low-cost solutions should also be considered as discussed in previous work in the field (Sharma & Saini, 2015; Rusitschka et al., 2009).

The NPT was used within this study as it has been shown to be able to inform implementation research and identify knowledge consistencies and gaps regarding implementation facilitators and barriers (Mair et al., 2012; O'Reilly et al., 2017), but it has not previously been used within a technology field, outside of healthcare-based technology. In addition, studies exploring the implementation of global health interventions have also not been widely supported using implementation theories and analytic frameworks (Ridde et al., 2020). However, recent work by Muchenje & Botha has highlighted the value of using theory when considering metering technology, as their study employed the theoretical framework: the technology acceptance model, to quantitively explore metering in South Africa (Muchenje & Botha, 2021).

The NPT demonstrated its value in this study. It was easily adopted for use within this study, allowing the factors affecting the implementation of metering to be thoughtfully considered. It did not extend to ‘fit’ all the findings seamlessly in the study, more specifically the findings around location and the context of metering. However, this was outweighed by its usefulness and application across the other findings, and building on from the study’s objectives, we offer a novel topic area within the implementation science field that the NPT can be applied to within a global health context.

Reflecting upon the limitations of our current study, the main area to highlight is the representativeness of our sample and hence the ability to generalise the findings across the wider population. As we used snowball sampling, our participants may therefore represent a group of individuals who have unique views on metering and is likely to include only those who are more willing or able to engage with research, especially as there were no incentives offered for participation. As the topic of metering and billing can be a sensitive area for participants, it could be deemed a limitation that we did not explore the influence of the researcher and their characteristics, which may have influenced the overall direction of the interview. Like other research projects in the same time frame, the COVID-19 pandemic affected research process and all interviews were forced to be conducted remotely.

## Conclusion

As a result of this work, we have been able to provide recommendations for future metering practice. The advantages of adopting smart metering systems includes energy saving and increased safety of supply and hence, recognising the value of this will be a key implementation facilitator. Other facilitators include meters being accessible to all residents and homeowners, regardless of their location. Metering technology needs to be practical and easy to understand, which can be ameliorated by adequate education around how to use a meter and how being aware of consumption can reduce bills. However, this will require greater social movement across the Global South. Awareness and education around metering technology needs to be simple and widespread, ensuring that those in lower socioeconomic groups are not left behind, and that they do not have to sacrifice potential earnings to spare time to educate themselves around learning how to use their utility meters. Finally, to ensure cost does not become an implementation barrier, future work should focus around making meters as cost efficient as possible, by taking the lead from other countries who have managed to achieve this effectively. This includes ensuring that the high costs associated with introducing new meters are not passed disproportionately onto the consumers and instead, they can be used as a tool to help reduce household expenditure by facilitating the implementation of tariff structures that would imply lower utility bills. Beneficial impacts of smart meters, such as controlling consumption, can also be realised when smart meters are coupled with home energy management technologies that assist residents in managing their electricity use from appliances.

## Supplementary Information

Below is the link to the electronic supplementary material.

Supplementary file1 (DOCX 25 kb)

Supplementary file2 (DOCX 27 kb)

## Abbreviations

HIG: High-income group
LIG: Low-income group
MIG: Middle-income group
NPT: Normalisation Process Theory
WP: Work Package

## Footnotes

1 For further information about the wider project, please refer to The British Academy website (The British Academy, 2022).
