# Supplementary material for: What is in a Meter? A Qualitative Exploration into the Implementation of Electricity Metering Across Mumbai Communities Using Normalisation Process Theory
Source: Glob Implement Res Appl. 2022 Oct 11;2(4):361–70. doi: 10.1007/s43477-022-00059-y (PMC9551251; doi:10.1007/s43477-022-00059-y)
Supplement: Supplementary file 1 — Supplementary file1 (DOCX 25 kb) [file 43477_2022_59_MOESM1_ESM.docx]

**Supplementary File 1.**

*Appendix 1: Interview Schedule*

*NOTE: The Interview schedules are developmental and questions may need to be tailored to the specific answers of each interviewee. As the interview process is designed to be iterative- the focus of questions may shift over the life of the fieldwork to better address the aims and objectives of the project or to provide additional information.*

1. Context Setting- *Identifying participant’s local area*

i) Could you please explain your current living arrangements, in as much detail as possible?

Prompts- location, type of accommodation and size, who do they live with e.g. family/ friends/shared, nearby facilities, services or resources, urban or rural, cost, are they happy or unhappy with it, any other thoughts or feelings.

ii) What do you believe to be the biggest advantages of your current living arrangements?

Prompts- Size, good access to services/resources, location, family support, cost, ease etc.

iii) What do you believe to be the biggest challenges to be with your current living arrangements?

Prompts- Size, poor access to services/resources, location, lack of family support, high cost etc.

iv) Generally speaking, what are the main demographics of the population in your area?

Prompts- socioeconomic status, gender, age, disability, ethnicity, race, religion etc.

v) What do you believe to be the biggest issues within your area?

Prompts- poor access to services/resources, employment, deprivation, high costs, crime, public health issues, disability etc.

2. Current Metering Access- *Exploring the participant’s current access and knowledge of the metering technology in their area*

vi) What do you know about metering technology e.g. water, electricity in your local area?

Prompts- nothing, a bit, a great deal, everything, access to information is good/bad etc, for each of the technologies

vii) What is your current access to metering technology e.g. water, electricity in your living arrangements?

Prompts- poor, limited, average, good, excellent, happy with it/unhappy with it

ix) What do you think are the facilitators to achieving or maintaining (depending on answer to vii) widespread metering in your area?

Prompts- cost, resources, location, policy making, employment, support

viii) What do you think are the barriers to achieving or maintaining (depending on answer to vii) widespread metering in your area?

Prompts- cost, socioeconomic status, resources, location, policy making, employment, disability

3. Cost of the current metering access- *Exploring the participant’s thoughts around the cost of metering technology in their area*

x) What do you think about the current cost of metering (water and electricity) in your area?

Prompts- fine, just right, too expensive, not sure

xi) Do you think the cost of services can act as a barrier to accessing metering in your local area? And if yes/no, why?

Prompts- accessible, poverty, deprivation, high costs?

4. Future Metering Access- *Exploring how the participant would like to maintain/ improve their access and knowledge of the metering technology in their area*

xii) How do you think the knowledge around metering technology in your area could be improved to become accessible to more of the population? (If at all)

Prompts- more widespread, increased knowledge, access, support to vulnerable groups, education, simpler

xiii) How do you think the metering technology in your area could be improved to become accessible to more of the population? (If at all)

Prompts- cheaper, more widespread, increased knowledge, access, education, policy support
