# Supplementary material for: What is in a Meter? A Qualitative Exploration into the Implementation of Electricity Metering Across Mumbai Communities Using Normalisation Process Theory
Source: Glob Implement Res Appl. 2022 Oct 11;2(4):361–70. doi: 10.1007/s43477-022-00059-y (PMC9551251; doi:10.1007/s43477-022-00059-y)
Supplement: Supplementary file 2 — Supplementary file2 (DOCX 27 kb) [file 43477_2022_59_MOESM2_ESM.docx]

**Supplementary File 2.**

*Appendix 2: Table of Codes by NPT Construct*

| Table 2 | | | | |
| --- | --- | --- | --- | --- |
| NPT  Construct |  | Subtheme |  | Code |
| Coherence | | Participants being able to see value to living arrangements | | spacious flat |
|  |  |  |  | calm environment |
|  |  |  |  | quiet environment |
|  |  |  |  | reasonable cost |
|  |  |  |  | near to work |
|  |  |  |  | good location |
|  |  |  |  | freedom |
|  |  |  |  | no challenges to living there |
|  |  |  |  | close to market/ services |
|  |  |  |  | no religious disputes/ segregation |
|  |  |  |  | youthful location |
|  |  |  |  | good transport links |
|  |  |  |  | secure building |
|  |  |  |  | ventilated/airy building |
|  |  |  |  | good owner |
|  |  |  |  | good schools |
|  |  |  |  | no issues with electricity |
|  |  |  |  | good/accessible services (compared to other areas) |
|  |  |  |  | nice area |
|  |  | Participants not seeing value to living arrangements | | rat problem |
|  |  |  |  | expensive or limited access to hospitals or health systems |
|  |  |  |  | shared toilets in poorer areas |
|  |  |  |  | limited furniture |
|  |  |  |  | leaking roof |
|  |  |  |  | mice |
|  |  |  |  | economic divide |
|  |  |  |  | water-logged sewage |
|  |  |  |  | busy roads |
|  |  |  |  | high accommodation/ rental cost |
|  |  |  |  | high population density |
|  |  |  |  | expensive electricity in area |
|  |  | Seeing the value in electricity and water | | understanding importance of electricity |
|  |  | Understanding the difference between old metering practice and new metering practice | | meters changed from mechanical to electrical |
|  |  |  |  | believing meters are more expensive- relying on older methods |
|  |  |  |  | lack of understanding of new technology |
|  |  |  |  | unsure if the cost varies between metering and old service |
|  |  | Area demographics | | mixed populations |
|  |  |  |  | socioeconomic group |
|  |  |  |  | urban area vs rural area |
|  |  |  |  | higher cost due to good facilities |
| Cognitive Participation | | Support/ Buy in to metering | | meters are open/ accessible |
|  |  |  |  | can monitor/access consumption easily |
|  |  |  |  | positive about change to electric meters |
|  |  |  |  | easy to understand |
|  |  |  |  | good availability |
|  |  |  |  | good communication about issues |
|  |  |  |  | supporting responsible usage |
|  |  |  |  | avoiding wastage |
|  |  |  |  | good enough |
|  |  |  |  | high cost not an issue of metering |
|  |  |  |  | accurate readings |
|  |  |  |  | price is reasonable |
|  |  | Negatives/ lack of support of metering | | higher price |
|  |  |  |  | paying for cost of meter |
|  |  |  |  | time |
|  |  |  |  | conflicts with leaders |
|  |  |  |  | limited access to meters |
|  |  |  |  | need online access for documents |
|  |  |  |  | not being aware of meters |
|  |  | Participants perform tasks as required | | can monitor/access consumption |
|  |  |  |  | easy to understand |
|  |  |  |  | others may lack understanding of technology |
|  |  |  |  | doesn’t check meter reading- checks bill |
|  |  |  |  | being too busy to monitor meter |
|  |  |  |  | unsure of what metering is in place |
|  |  |  |  | not knowing where meter is |
|  |  |  |  | misinformation from leaders |
|  |  |  |  | pleased with way society is doing it |
|  |  |  |  | doesn’t have much to do with meter- society/ security controlled |
|  |  |  |  | doesn’t know meter reading |
| Collective Action | | Supporting the general practice of metering | | awareness of technology being positive |
|  |  |  |  | majority of population are aware of meters |
|  |  |  |  | easy to do meter readings |
|  |  | Not supporting/ issues with the general practice of metering | | issues/complaints with energy meters |
|  |  |  |  | meter damage |
|  |  |  |  | not accurate readings |
|  |  |  |  | not interested in meter |
|  |  |  |  | not being able to access meters in accommodation |
|  |  |  |  | no metering in slums |
|  |  |  |  | negative public opinion on meters (due to them believing it will cost more) |
|  |  |  |  | political reasons preventing metering |
|  |  |  |  | differing goals |
|  |  |  |  | expense of switching utility companies |
|  |  |  |  | corruption of utilities |
|  |  |  |  | not being able to take care/ maintain meters in slums |
|  |  |  |  | tampering with energy meters |
|  |  |  |  | security deposits inaccessible to low income groups |
|  |  |  |  | problematic getting documents |
|  |  |  |  | COVID-19 lockdown meaning readings aren’t being taken |
|  |  |  |  | need online access for documents |
|  |  |  |  | issues with literacy |
| Reflexive Monitoring | | How to improve future practice | | unsure of area’s/ other areas access |
|  |  |  |  | using WhatsApp/ phones |
|  |  |  |  | exploring ways of monitoring/controlling consumption |
|  |  |  |  | installing limits on usage |
|  |  |  |  | information sharing |
|  |  |  |  | focusing on socio-political issues |
|  |  |  |  | speaking out about metering not being more expensive |
|  |  |  |  | need to reach more people/ better awareness |
|  |  |  |  | better service and confidence in utility providers |
|  |  |  |  | support with security deposits |
|  |  |  |  | helping people with literacy issues/ no internet |
|  |  |  |  | making the process more automatic/ integrating automatic system |
|  |  |  |  | making it a requirement |
|  |  |  |  | need improvements in technology |
|  |  |  |  | maintenance of meters |
|  |  |  |  | Improvements need to be made to meter technology (less impact/ better maintenance) |
|  |  |  |  | customers being aware of consumption |
|  |  | Participants accessing information about metering technology | | unsure of other areas access |
